# On-Screen Visual Feedback Effect on Static Balance Assessment with Perturbations

**DOI:** 10.3390/s24051588

**Published:** 2024-02-29

**Authors:** Ruben Valenzuela, Javier Corral, Mikel Diez, Francisco J. Campa, Saioa Herrero, Erik Macho, Charles Pinto

**Affiliations:** Bilbao School of Engineering, University of the Basque Country UPV/EHU, 48013 Bilbao, Spain; j.corral@ehu.eus (J.C.); mikel.diez@ehu.eus (M.D.); fran.campa@ehu.eus (F.J.C.); saioa.herrero@ehu.eus (S.H.); erik.macho@ehu.eus (E.M.); charles.pinto@ehu.eus (C.P.)

**Keywords:** balance assessment, parallel manipulator, force plate, posturography, centre of pressure

## Abstract

In this study, the novel mobile dynamometric platform, OREKA, was utilized to perform an extensive analysis of the centre of pressure behaviour during different tilt motion exercises. This platform is based on a parallel manipulator mechanism and can perform rotations around both horizontal axes and a vertical translation. A group of participants took part in an experimental campaign involving the completion of a set of exercises. The aim was to evaluate the platform’s potential practical application and investigate the impact of visual on-screen feedback on centre of pressure motion through multiple balance indicators. The use of the OREKA platform enables the study of the impact on a user’s balance control behaviour under different rotational perturbations, depending on the availability of real-time visual feedback on a screen. Furthermore, it presented data identifying postural control variations among clinically healthy individuals. These findings are fundamental to comprehending the dynamics of body balance. Further investigation is needed to explore these initial findings and fully unlock the potential of the OREKA platform for balance assessment methodologies.

## 1. Introduction

Body balance is essential for performing everyday tasks such as walking, running, or standing, and allows individuals to maintain autonomy in their daily activities [1]. To achieve this, humans acquire sensorial inputs obtained from the vestibular, visual, and somatosensory systems which are then processed by the central nervous system (CNS) to produce the required muscular activity to maintain the vertical projection of the centre of mass (CoM) within the limits of the base of support (BoS) [2]. The CoM is defined as the point where all the mass of the human body can be considered concentrated, and the BoS is the area delimited by the placement of the feet [3].

Body balance can be categorized as static or dynamic. Static balance refers to maintaining the CoM in a specific posture without disturbances, whereas dynamic balance involves projecting the CoM outside the BoS, like walking or moving from sitting to standing [4]. Numerous studies have consistently demonstrated that falls are predominantly associated with dynamic balance rather than static balance [5].

Unfortunately, balance impairment is common, affecting the quality of life of several people. One of the most common pathologies that affects balance is cerebrovascular disease (CVD) or stroke. In Spain, around 890 cases were recorded annually between 2010 and 2014 [6]. After stroke, it becomes crucial to assess the degree of impairment in mobility and balance [7]. To achieve this, several types of standardized clinical evaluations have been developed.

One of the most frequently applied tests to assess balance is the Romberg test [8]. In this evaluation, the patient is requested to maintain balance during a standing position and walking with two conditions: eyes open (EO) and eyes closed (EC). The premise is that when the vision sensorial input is absent, the remaining proprioceptive and somatosensory systems are sufficient to keep balance [9]. If a significant sway is present during the EC condition, the doctor diagnoses potential issues with the proprioceptive and somatosensory systems. The main issue with this test is that the “significant sway” is entirely subjective to the doctor that is assessing the patient.

Other evaluations like the Berg Balance Scale (BBS) were originally designed to assess balance in older adults [10], and their use was later extended to assess stroke patients [11]. This test consists of 14 functional tasks divided into three categories: sitting balance, standing balance, and dynamic balance. These are performed in a clinical setting, and a score is assigned based on the time it takes the user to complete the task or perform it without the need for any kind of assistance, taking approximately 10 to 15 min to complete. Each item is then assigned a score between 0 and 4 points for a total global score of 56 points. Scores ranging from 0 to 20 points indicate a balance impairment, 21 to 40 points represent an acceptable balance, and 41 to 56 a good balance.

Another test that evaluates the performance of specific tasks is the Tinetti Balance Test (TBT); in detail, this assessment is divided into static and dynamic balance, for example, standing with both feet together or on one leg, sitting and standing off a chair, and gait analysis, initiation of gait, step height, step length, etc. [12]. After a task is completed, a score of 0, 1, or 2 is assigned depending on the performance shown by the user, where the maximum possible score is 44 points. For example, when evaluating rising from a chair, the three possible performances are as follows: Unable to stand without help (score = 0), able to stand but uses arms to help (score = 1), and able without use of arms (score = 2). After completing all the tasks and adding up the scores, if the total score is below 18 points, it is considered that the person has a high fall risk, if the score is between 19 and 23 it relates to a moderate fall risk, and if the score is above 24 then a low fall risk is assigned. This test was originally planned to be applied to older adults, but nowadays it can be applied to any patient deemed to possess fall risk [13]. It is important to note that some of the test considerations used to evaluate the score involve a highly subjective assessment, e.g., when evaluating the eyes closed standing posture, if the subject is unstable a score of 0 is assigned, whereas if the subject is stable a score of 1 is assigned, but what is judged as stable may vary for different doctors.

There are also other types of clinical assessment mobility tests that require less equipment and time to perform, such as the Timed Up and Go (TUG) test which can also be used to identify balance issues [14]. This test consists of measuring the time it takes to stand up from a chair, walk 3 m, turn back, and sit in the chair again. If the time it takes to perform this task is less than 20 s then the subject is considered to have a normal mobility, if the score is between 21 and 29 s then the subject is considered to have some mobility issues and may need assistance, and if the length is greater than 30 s then the subject is considered to be completely dependent.

Tests have also been developed to specifically evaluate people who have had a stroke, such as the Rivermead Motor Assessment (RMI). This test consists of a questionnaire with 14 binary (Yes/No) questions related to the person’s autonomy to perform routine activities and direct observation of the user getting up from a chair without assistance [15]. The questions are ordered from least to most difficult, and each one is assigned a score of 0 or 1 depending on the response, so the highest possible score is 15 points.

The clinical tests mentioned above involve two distinct methodologies. The first method measures the time it takes for a patient to execute a specific task and the second one evaluates how the task is performed. While these tests provide valuable insights into balance assessment, they also possess a high degree of subjectivity that could affect their accuracy. To solve this subjectivity problem, a second technique known as posturography can be used, which employs quantitative measurements for balance assessment [16,17,18,19,20,21,22,23].

This method, grounded in measurable, precise, and repeatable indicators, relies heavily on indices such as those derived from the centre of mass (CoM) or the centre of pressure (CoP), defined as the point in the ground where a single applied force would have the same translational and rotational effects as the actual distributed applied forces. The indices of CoP, such as movement amplitude and speed, are widely acknowledged to be related to the balance function [24]. These movement parameters can provide a relevant insight into an individual’s postural control, with distinct patterns being observable between healthy subjects and those with compromised balance. Given that obtaining the behaviour of the CoM in real time can be time-consuming, as it requires either measuring the weight of each body segment and its position in 3D space or the video input of 3D kinematic models, the use of CoP is often preferred [21].

Posturography paradigms can be classified into static or dynamic [25,26,27], with each one being able to evaluate different postural control mechanisms. These paradigms serve as references from which measurements, observation, and analysis of the patterns and movements can be associated with balance and posture.

The static posturography paradigm focuses on an individual’s ability to maintain a steady standing posture on a flat, horizontal surface under various conditions, such as EO or EC [28,29]. This paradigm is particularly significant in identifying how the body uses ankle torque to stabilize the CoM within the boundaries of the BoS, thereby maintaining balance with minimal deviations [17].

On the other hand, the dynamic posturography paradigm introduces deliberate perturbations to assess the individual’s adaptive responses within their postural control system [30,31,32]. This enables a thorough examination of the body’s reflexes and responses to unexpected changes, which is critical in real-world environments where balance is constantly challenged.

The CoP signal obtained from these paradigms offers important insights into the complexity of the postural control mechanism and the potential changes brought about by pathological conditions. The indices derived from the CoP signal such as mean distance, mean velocity, and prediction ellipse area, among others, provide a quantitative basis for analysing body sway, balance, and postural stability [33,34].

There are studies indicating differences in postural control behaviour among users based on whether or not they receive visual feedback on a screen [35]. These observations, however, are primarily based on experiments that were conducted using static dynamometric platforms.

The aim of this paper is to explore variations in the behaviour of different CoP indices using the mobile platform OREKA, a novel mobile dynamometric platform designed and developed at the University of the Basque Country for balance evaluation. Our goal is to explore the impact of real-time visual feedback on a screen on balance indicators among clinically healthy individuals.

## 2. Materials and Methods

### 2.1. Description of the OREKA Platform

**Figure 1:**
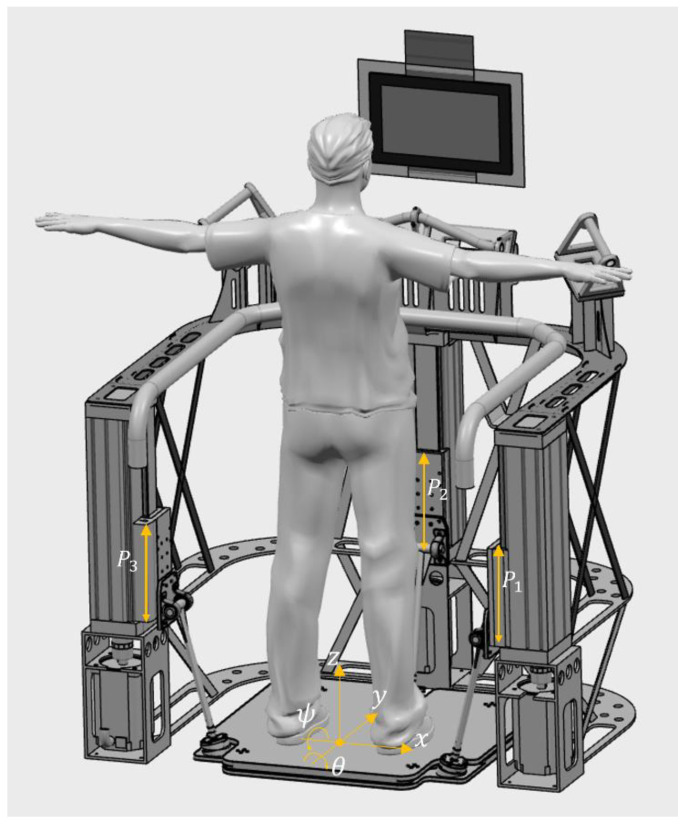
OREKA platform ($P_{n}$ where n=1…3 are the prismatic joints).

The OREKA (balance in Basque) dynamometric mobile platform is based on a three-kinematic-chains prismatic-revolute-spherical (PRS) joint parallel mechanism with three degrees of freedom (Figure 1). In a coordinate system where *x*-axis represents left/right, *y*-axis represents forward/backward, and *z*-axis represents up/down, the platform can perform rotations around the *y* and *x* axis, roll/mediolateral (ϴ), and pitch/anteroposterior (ψ), respectively, and a displacement on the *z*-axis. The platform is actuated by the vertical prismatic joint $P_{n}$, where n=1…3 with a travel of 250 mm. The pitch and roll angle ranges were determined based on the requirements provided by rehabilitation medical specialists, allowing for a range of ±15° for both variables. Displacement in the z direction is achieved by simultaneously activating all three actuators in the same direction.

The mobile platform consists of two aluminium plates with a non-uniform thickness of at least 12 mm, with reinforcement ribs up to a maximum thickness of 20 mm. The bottom plate is where the spherical joints of the PRS mechanism are located. In between the plates, four uniaxial force sensors have been placed at the corners to acquire force signals to be used to compute the position. The top plate provides the surface on which the subject stands during testing.

The OREKA platform’s hardware consists of three synchronous motors chosen for this setup, each with a power of 3.6 kW and identified as B&R (www.br-automation.com, B&R: Eggelsberg, Austria) model 8LSA55.DA030S000-3. These motors are governed by ACOPOS P3 controllers, specified as model 8EI013HWS10.0200-1 (B&R). They are integrated with ball bearing linear guides (model EGC-BS-KF-GK) from FESTO (www.festo.com, FESTO: Esslingen, Germany). Motion control is performed through the B&R panel PC 3100. Additionally, the system employs four uniaxial force sensors—Interface GWMC (www.interfaceforce.com, Interface Force: Scottsdale, AR, USA)—employing strain gauges capable of measuring forces up to 2000 N. These sensors are connected to X20AI4636 B&R modules for data acquisition, capturing information at a frequency of 50 Hz. The OREKA platform is patented with the reference number WO 2023/052670 [36].

**Figure 2:**
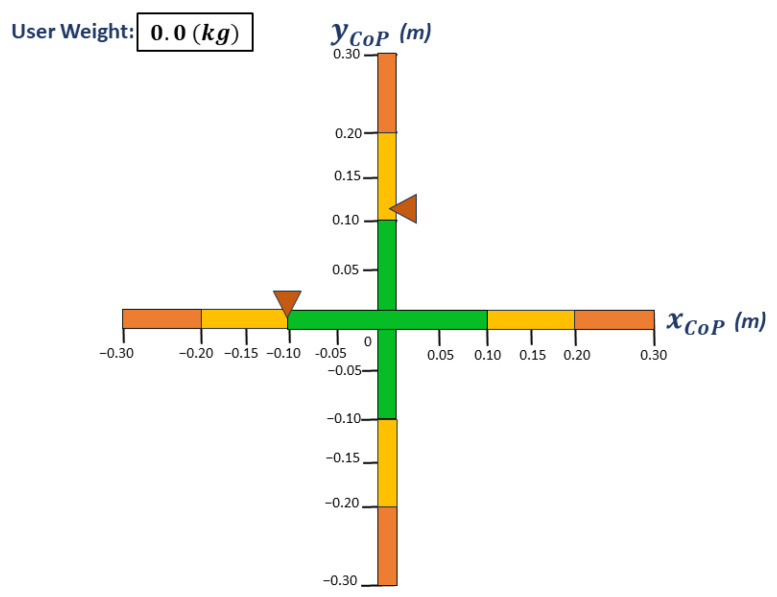
Visual feedback screen. The triangles are the markers that indicate both xCoP and yCoP positions.

The OREKA platform is designed to provide real-time feedback on the position of the CoP to the user while the platform rotations are ongoing. This is achieved by visually displaying the AP and ML position on a screen (Figure 2). If the user leans forward, then the marker corresponding to the yCoP (AP direction of the CoP) moves upwards; conversely, if the user leans backwards, then the marker moves downwards. Similarly, in the xCoP (ML direction of the CoP) direction, if the user leans to the right, then the marker also moves to the right, and finally if the user leans to the left, then the marker moves to the same left direction.

### 2.2. Experimental Protocol

For this study, twenty-eight healthy young adults were recruited, all of whom willingly participated and informed consent was obtained. The participants, a combination of teachers and students, were all from the University of the Basque Country UPV/EHU. Table 1 presents a detailed overview of their respective characteristics.

The OREKA platform can rotate in the anteroposterior (AP) and mediolateral (ML) directions, which can vary in amplitude and angular velocity. Each exercise has a duration of 1 min. For this study, a selection of nine exercises (Table 2) were performed to maintain the test duration below 25 min to avoid fatigue [37].

**Figure 3:**
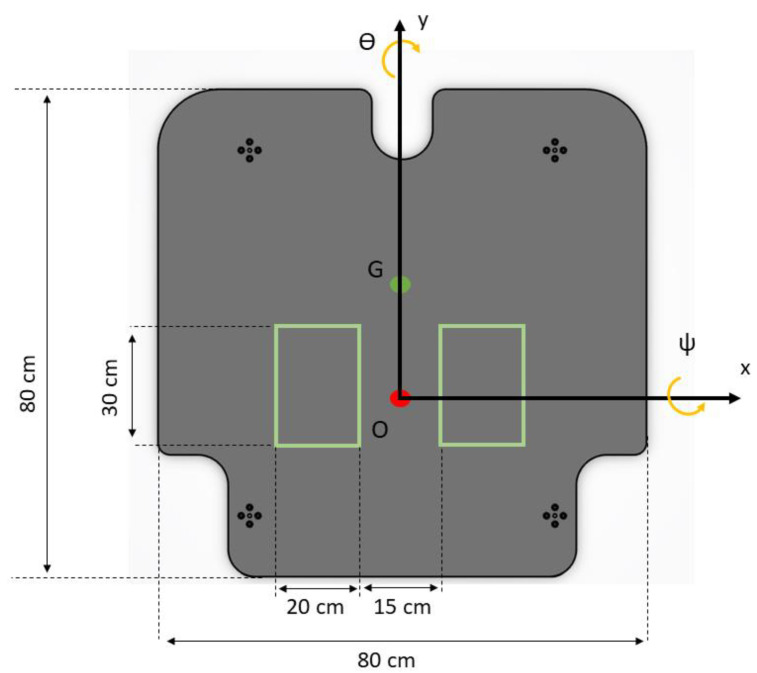
Platform dimensions and area for feet placement.

To ensure repeatability and to maintain a uniform CoP reference, the users were instructed to place their feet within a predefined area that would allow them to stand shoulder width apart, aligning their ankles with the *x* axis (Figure 3). Their arms were to fall naturally at their sides, and they were to always maintain an upright posture.

For tests with feedback, a screen was positioned 1.5 m away. Users were instructed to keep the markers shown on the screen (see Figure 2) as steady as possible in the initial position after they had stood within the marked area. To achieve this, they could make the necessary joint movements without lifting their feet off the platform.

For tests without feedback, the screen was replaced with a black marker positioned at the same distance. Users were instructed to keep their gaze fixed on this marker and maintain their back in an upright posture as steady as possible, using whatever natural strategies their bodies required to achieve this goal.

### 2.3. Data Processing

As stated before, our system uses four uniaxial sensors, Interface GWMC, positioned at the corners between the two plates. These sensors measure the reactive forces produced by a user’s weight at a sampling frequency of 50 Hz. Before interpreting these data, a conditioning stage to filter out any unwanted signals that could distort the calculation of the CoP behaviour must be applied [38].

**Figure 4:**
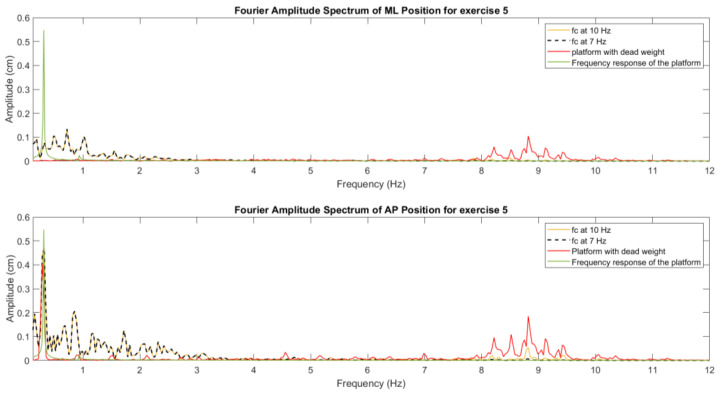
Amplitude spectrum behaviour of the measurements with different cut-off frequencies for the filter.

It is worth noting that 99% of the frequency spectrum of the CoP signal ranges from 0–6.5 Hz [39]. During the first experimental trials, we detected that the OREKA platform’s own kinematic movement introduced some perturbances at higher frequencies. Therefore, to attenuate the influence of the platform kinematics on the recorded CoP signal, a zero-phase Butterworth filter with a cut-off frequency located at 7 Hz was applied. Given the platform’s maximum cycle frequency of 1 Hz, the majority of spectral power of the CoP signal would tend to be concentrated in the range of 0–1 Hz. With this, the vibratory effect on the computed indices and the high frequency noise was minimized. In Figure 4, the Fourier amplitude spectrum with different filters can be seen, as well as the platform spectrum with a dead weight and the frequency response of the movement of the platform. It can be noticed that the filter at 7 Hz removes the noise caused by the platform kinematics, maintaining the overall information at lower frequencies.

When computing the velocity of the CoP ($v_{CoPx}$, $v_{CoPy}$), a differential algorithm to both the x and y coordinates of the CoP positions was applied. However, this process amplifies some high-frequency components in the velocities; thus, a second conditioning stage is necessary. In this case, a Savitsky–Golay filter is applied. This filter was selected for its characteristic to preserve important data features while smoothing out noise. To obtain a cut-off frequency close to 10 Hz, a combination of parameters is required: a fourth-order filter with a sampling window of 17 elements.

### 2.4. Inertial Compensation

When assessing balance on a moving platform, it is important to consider the inertia effects of the upper plate on the sensors. This factor can influence the measurements of the CoP. Therefore, incorporating a compensation mechanism in the data post-processing stage is essential to ensure accuracy in the results.

As previously stated, the rotations are performed around the *x* and *y* axis to produce both AP (ψ) and ML (ϴ) rotations, respectively. The fixed reference frame OXYZ and the platform reference frame xyz are placed in the same position (Figure 3) 2 mm below the upper plate plane, aligning with the horizontal plane that contains the centre of the three spherical joints. The rotation matrix R used to specify the rotations performed in the platform reference frame xyz with respect to the fixed reference frame OXYZ is as follows:

(1) $$R=R_{y}R_{x}=\left[\begin{array}{ccc} cos\theta & sin\psi sin\theta & cos\psi sin\theta \\ 0 & cos\psi & -sin\psi \\ -sin\theta & sin\psi cos\theta & cos\psi cos\theta \end{array}\right]$$

**Figure 5:**
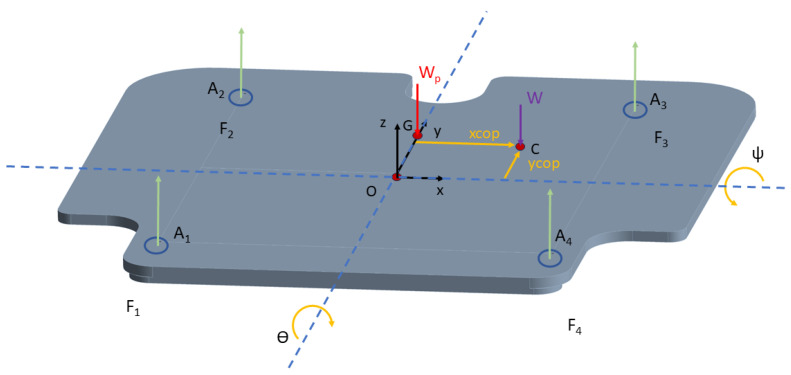
Free body diagram of the platform.

Applying Newton’s second law along the *z* axis of the platform frame, the equation with the forces shown in Figure 5 is as follows:

(2) $$F_{1}+F_{2}+F_{3}+F_{4}-W_{p}cos\psi cos\theta -Wcos\psi cos\theta =Ma_{Gz}$$

where

$F_{i};\;i=1\ldots 4$ are the forces measured by the sensors.
$W_{p};$ The weight of the platform.
W; The weight of the user.

The projection of the weight W on the platform reference frame is then obtained as follows:

(3) $$W=\left\{\begin{array}{c} W_{x}\\ W_{y}\\ W_{z} \end{array}\right\}=R^{T}\left\{\begin{array}{c} 0\\ 0\\ -W \end{array}\right\}=\left[\begin{array}{ccc} cos\theta & 0 & -sin\theta \\ sin\psi sin\theta & cos\psi & sin\psi cos\theta \\ cos\psi sin\theta & -sin\psi & cos\psi cos\theta \end{array}\right]\left\{\begin{array}{c} 0\\ 0\\ -W \end{array}\right\}=\left\{\begin{array}{c} Wsin\theta \\ -Wsin\psi cos\theta \\ -Wcos\psi cos\theta \end{array}\right\}$$

Reordering Equation (2), the forces that the sensors are measuring are as follows:

(4) $$F_{1}+F_{2}+F_{3}+F_{4}=Ma_{Gz}+W_{p}cos\psi cos\theta +Wcos\psi cos\theta$$

To compensate the moments around the *X* and *Y* axis, Newton’s second law for rotational systems with respect to G is applied:

(5) $$N_{G}=N_{GW}+N_{GFi}=\frac{dH_{G}}{dt}$$

where $N_{G}$ is the total torque and $\frac{dH_{G}}{dt}$ is the rate of change in the angular momentum.

$\frac{dH_{G}}{dt}$ is obtained analytically through the formula $\frac{d}{dt}\left(I_{G}\times \omega \right)$, where $I_{G}$ represents the moment of inertia and ω the angular velocity. To implement this formula, we used the data acquired by the platform, which include measurements of rotational velocity and acceleration. Additionally, the moment of inertia $I_{G}$ and the inertia products are obtained through the CAD model of the platform.

Since only the weight of the user and the force on the sensors exert a torque on the platform, each torque is calculated as follows:

(6) $$N_{GW}=GC\times W=\left\{\begin{array}{c} {GC}_{y}W_{z}-{GC}_{z}W_{y}\\ {GC}_{z}W_{x}-{GC}_{x}W_{z}\\ {GC}_{x}W_{y}-{GC}_{y}W_{x} \end{array}\right\}$$

and **GC** is defined as

(7) $$GC=\left\{\begin{array}{c} {GC}_{x}\\ {GC}_{y}\\ {GC}_{z} \end{array}\right\}=GO+OC=-OG+OC=\left\{\begin{array}{c} -{OG}_{x}+x_{COP}\\ -{OG}_{y}+y_{COP}\\ -{OG}_{z}+z_{COP} \end{array}\right\}$$

(8) $$N_{GFi}={GA}_{i}\times F_{i}=\left\{\begin{array}{c} {GA}_{iy}F_{iz}\\ {-GA}_{ix}F_{iz}\\ 0 \end{array}\right\}$$

and **GA** is defined as

(9) $$GA_{i}=GO+OA_{i}=-OG+OA_{i}$$

(10) $$GA_{1}=\left\{\begin{array}{c} -{OG}_{x}-a\\ -{OG}_{y}-b\\ -{OG}_{z}+z_{COP} \end{array}\right\}$$

(11) $$GA_{2}=\left\{\begin{array}{c} -{OG}_{x}-a\\ -{OG}_{y}+b\\ -{OG}_{z}+z_{COP} \end{array}\right\}$$

(12) $$GA_{3}=\left\{\begin{array}{c} -{OG}_{x}+a\\ -{OG}_{y}+b\\ -{OG}_{z}+z_{COP} \end{array}\right\}$$

(13) $$GA_{4}=\left\{\begin{array}{c} -{OG}_{x}+a\\ -{OG}_{y}-b\\ -{OG}_{z}+z_{COP} \end{array}\right\}$$

where

a; Distance in *x* axis from O to the sensor A_i_.
b; Distance in *y* axis from O to the sensor A_i_.

To calculate the xCoP and yCoP positions with the inertial compensation, the following equations are used:

(14) $$x_{CoP}={OG}_{x}-\frac{{\left(\frac{{dH}_{G}}{dt}\right)}_{y}+\sum_{i=1}^{4}{GA}_{ix}F_{i}-(-{OG}_{z}+z_{CoP})W_{x}}{W_{z}}$$

(15) $$y_{CoP}={OG}_{y}+\frac{{\left(\frac{{dH}_{G}}{dt}\right)}_{x}-\sum_{i=1}^{4}{GA}_{iy}F_{i}+(-{OG}_{z}+z_{CoP})W_{y}}{W_{z}}$$

where $z_{CoP}$ is 0.2 cm (sensors measuring point), ${OG}_{x}=0$, ${OG}_{y}=1.711$ cm, and ${OG}_{z}=-0.938\;cm$. These values were obtained with the CAD model of the platform.

### 2.5. CoP Indicators

To compute each indicator, we use a sample of 30 s, ranging from 10 to 40 s from the original minute duration of the exercise, to eliminate the variations present during the start and end of the rotation and to ensure the cyclic behaviour of a user during the exercise.

**Figure 6:**
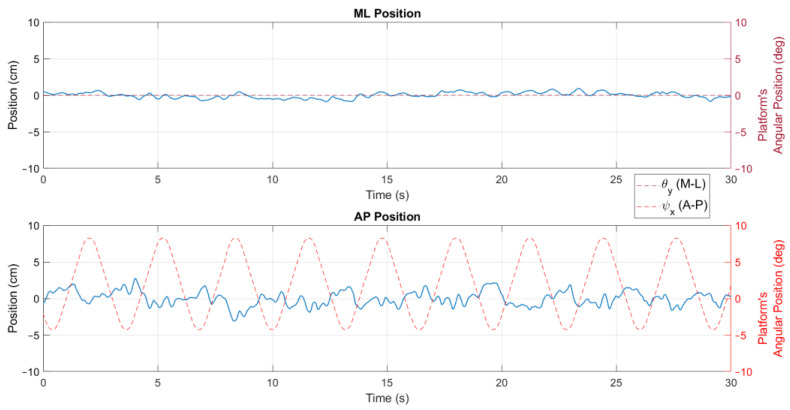
Position stabilogram of a user for exercise 5 ([−8°, +4°] AP; 10°/s) with visual feedback on a screen.

**Figure 7:**
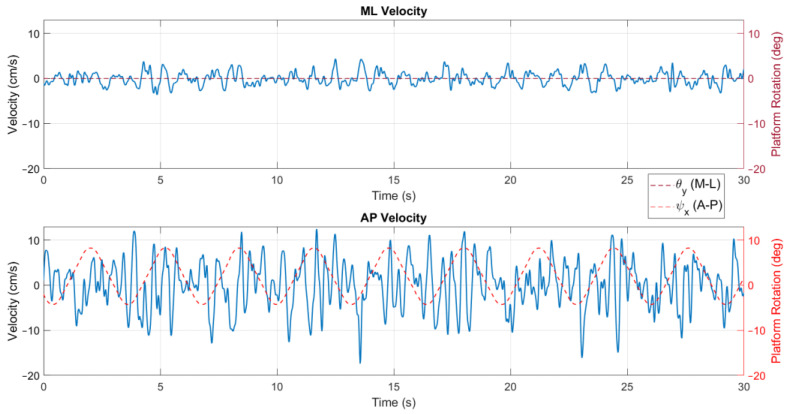
Velocity signal for exercise 5 ([−8°, +4°] AP; 10°/s) with visual feedback on a screen.

The position metrics offer insights into a user’s body sway and how it varies over time and across the different conditions of the tests. In Figure 6, a stabilogram that illustrates the AP and ML positions is presented, highlighting observable differences between these positions as influenced by the platform’s rotation. In addition, the velocity parameters describe the speed at which body sway occurs, reflecting the responsiveness and adaptability of a user’s postural control system to the platform’s movements, as shown in Figure 7.

**Figure 8:**
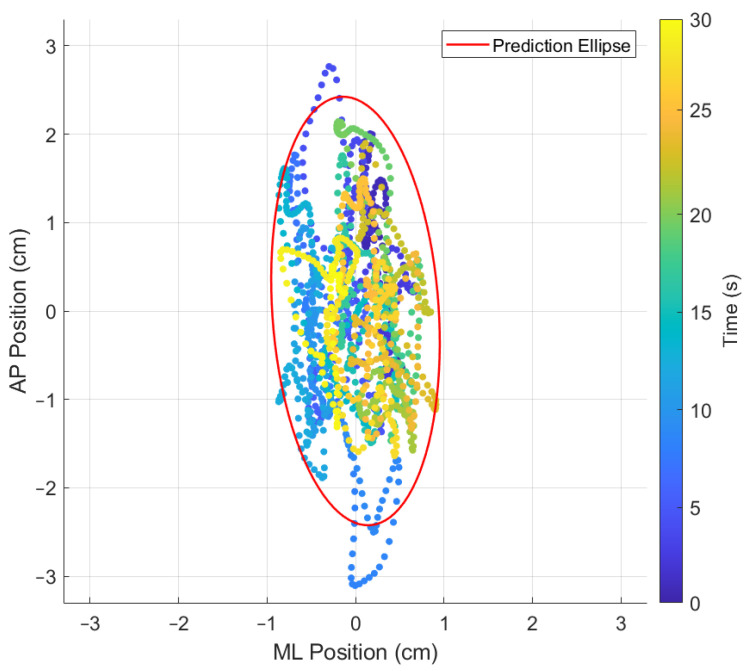
Statokinesigram for exercise 5 ([−8°, +4°] AP; 10°/s) with feedback on a screen.

The 95% prediction ellipse area (PEA) of the statokinesigram is determined by first calculating the sample variance in the ML and AP directions as well as the sample covariance between ML and AP, all of which are contained in the sample covariance matrix S (Table 3). Following this, the chi-squared value with two degrees of freedom, which will encompass a future data point with 95% of probability (represented as $\chi_{0.95,2}^{2}$), is identified using reference tables. The final step in computing the PEA involves taking the square root of the product of the two eigenvalues of [S] and multiplying this value by $\chi_{0.95,2}^{2}$ [40]. This indicator provides an overall measure of the body sway, offering a global view of the postural stability during each exercise. In Figure 8, a statokinesigram is shown where the evolution through time is depicted as a colour variation from cold (test start) to warm (test end) colours.

**Figure 9:**
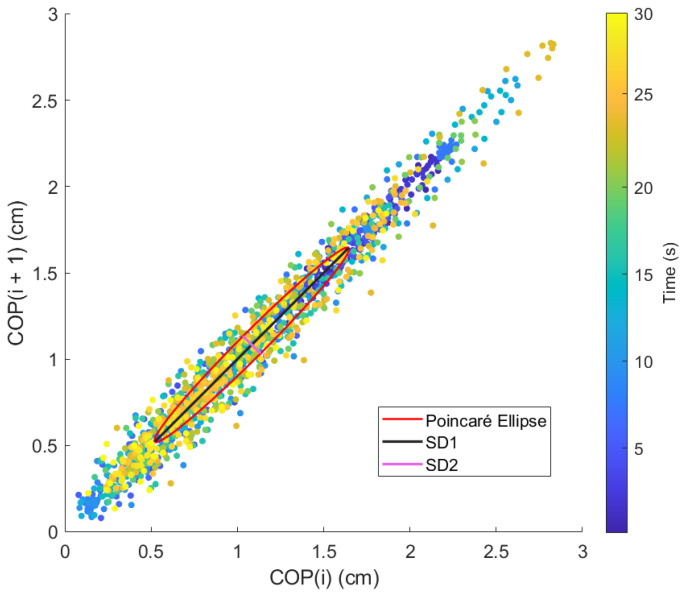
Poincaré diagram for exercise 5 ([−8°, +4°] AP; 10°/s) with feedback on a screen.

Lastly, the ellipse axis SD1/SD2 of the Poincaré diagram, where SD1 is the minor axis, perpendicular to the identity line, and SD2 is the major axis, parallel to the identity line (Figure 9), offers an analysis of the variability and the correlation between consecutive data points in the CoP signal [41]. This gives a unique insight into the control mechanisms used by the postural control system under the varying conditions presented by the OREKA platform.

Other research fields, like electrocardiogram analysis have applied the Poincaré plot as an assessment tool for assessing beat-to-beat variability. It displays a signal against itself with a delay, resulting in a scatter of points forming a cloud around a 45° line of identity. This cloud of points can be enclosed by an ellipse, which can be analysed to extract valuable information about the heart rate variability [42]. The major axis, the standard deviation along the line of identity of the Poincaré plot (SD2), corresponds to long-term variability, while the minor axis, the standard deviation perpendicular to the line of identity (SD1), represents short-term variability.

The use of parameters such as mean distance, mean velocity, prediction ellipse area of the statokinesigram, and the SD1/SD2 ellipse axis of the Poincaré diagram provides a significant role in describing postural control behaviour through various rotations and angular velocities performed by the OREKA platform (Table 3).

To compute the mean distance, mean velocity, the 95% PEA, and the Poincaré diagram, first, the datapoints must be centred to the origin. To do that, for every ${xCoP}_{i}$ and ${yCoP}_{i}$ measured point, the mean value of all the positions is then subtracted:

(16) $$\mu_{ML}=\frac{1}{N}\sum_{i=1}^{N}{xCoP}_{i}$$

(17) $$\mu_{AP}=\frac{1}{N}\sum_{i=1}^{N}{yCoP}_{i}$$

(18) $${CoP}_{xi}={xCoP}_{i}-\mu_{ML}$$

(19) $${CoP}_{yi}={yCoP}_{i}-\mu_{AP}$$

where N is the total number of datapoints. Once every $x_{CoPi}$ $y_{CoPi}$ are calculated, velocities are then obtained by applying a discrete centred differential algorithm:

(20) $$v_{CoPxi}=\frac{{CoP}_{x}\left(i+1\right)-{CoP}_{x}(i-1)}{t\left(i+1\right)-t(i-1)}$$

(21) $$v_{CoPyi}=\frac{{CoP}_{y}\left(i+1\right)-{CoP}_{y}(i-1)}{t\left(i+1\right)-t(i-1)}$$

where t is time. With these data, the indicators in Table 3 can be calculated.

Where S is the covariance matrix of the sample and $\chi_{0.95,2}^{2}=5.99146$ for a fixed probability level of P=95%. This gives a unique insight into the control mechanisms used by the postural control system under the varying conditions presented by the OREKA platform.

### 2.6. Data Analysis

The normality of the distribution was verified using the Shapiro–Wilk test given the presence of a normal distribution of quantitative variables and the use of parametric statistics. Therefore, a paired T-test for comparing means was applied. The α level for all statistical tests was set at 0.05. In each of the CoP indices, a table is provided with the comparison of the mean values of the tests with on-screen feedback with respect to the tests without on-screen feedback, along with the respective *p*-values.

## 3. Results and Discussion

### 3.1. Characterization of x and y Distances

**Figure 10:**
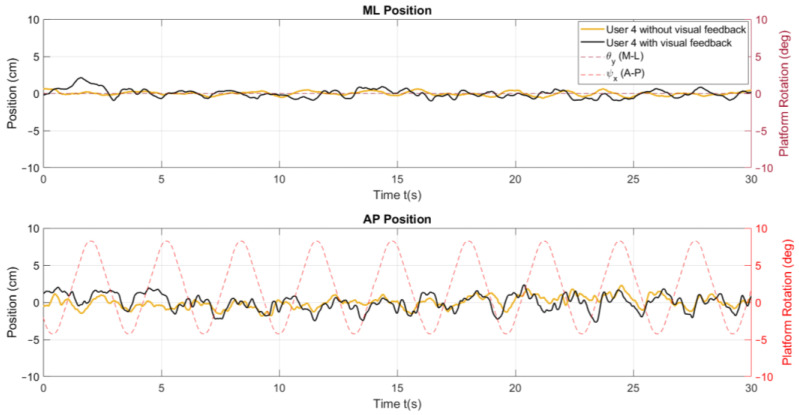
Comparison of the ML and AP position behaviours for exercise 5 ([−8°, +4°] AP; 10°/s).

In Figure 10, variations in AP and ML positions are observed between the same user by modifying the test conditions with and without real-time visual feedback on a screen, even though they are classified as clinically healthy. When feedback on a screen is present, a wider amplitude of the signal, compared to the amplitude of the signal without said feedback, can be observed.

An explanation for this is the change in the task requirements for the users. Initially, with feedback on a screen, the users were requested to keep the markers fixed at the initial position, a task that demands active engagement and continual adjustments to maintain balance. This scenario inherently causes more significant CoP displacements as the users actively respond to the feedback on a screen to manage their postural control.

When feedback on a screen is removed, the nature of the task undergoes modification. Now, the users are only required to maintain an upright stance, with no specific targets for alignment. Under these conditions, their need to actively respond to visual cues is removed, allowing the users to fall back on a more passive postural control strategy that likely corresponds more closely to their natural body sway.

In Table 4, which presents the mean and standard deviations of the x and y distances, there is a reduction in these values when feedback on a screen is removed. As previously stated, this reduction could be attributed to the user changing from actively maintaining balance to instinctively adapting to the platform’s rotation using natural body responses when the task of keeping the markers at their initial position is removed. The decrease in the standard deviation further supports this interpretation. It suggests that users adapt more effectively to the platform’s rotations without feedback on a screen.

It is also worth noting that when the platform rotates in the AP direction, the CoP motion in the x direction is smaller than the y direction when the platform rotates in the ML direction. This difference may be due to the user’s confidence in controlling balance in the x direction. This is a regularly engaged movement pattern, for example, when shifting the body weight between legs during extended periods while on a standing posture. On the other hand, swaying in the y direction is less common in typical movements, making it a less familiar balance challenge to navigate.

Table 5 highlights that exercises 2, 3, and 9 present a significant increase (*p* < 0.05) in the $d_{{CoP}_{x}}$. Similarly, in the $d_{{CoP}_{y}}$, it is shown that exercises 3 and 7 exhibit this significant increase in the mean values (*p* < 0.05).

### 3.2. Characterization of x and y Velocities

**Figure 11:**
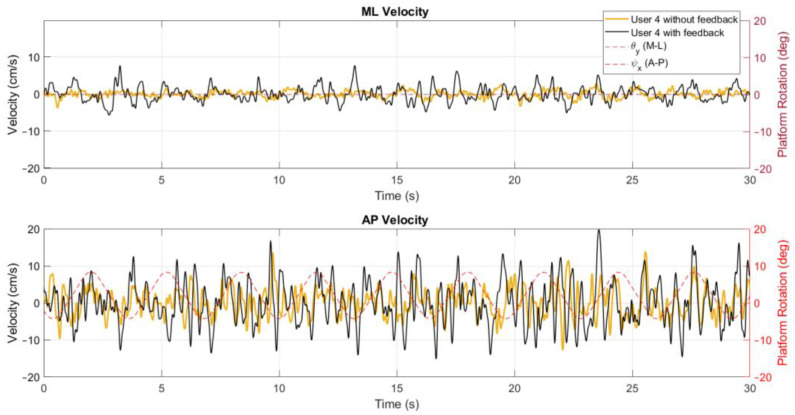
Comparison of the $v_{CoPx}$ and $v_{CoPy}$ behaviour for exercise 5 ([−8°, +4°] AP; 10°/s).

When comparing the exercises in conditions with and without on-screen feedback in the velocity behaviour, a similar conclusion to the one of the positions can be drawn (Figure 11). Considering that without feedback on a screen the user does not need to actively adjust to the platform rotation, the velocity of the CoP signal is significantly lower, meaning that the user’s adaptive balance control is already acquainted to the platform movement.

As shown in Table 6, a similar trend is seen in the mean and standard deviations in $v_{CoPx}$ and $v_{CoPy}$. This reduction when the feedback on a screen is removed further solidifies the theory that there exists a transition from an active to a more adaptive postural control strategy. This last approach, characterized by instinctive body adjustments, reduces the need for rapid movements.

In the rotations where the amplitude was low, the increment of the $v_{CoPx}$ with the addition of the on-screen visual feedback is significant (*p* < 0.05), as stated in Table 7, for exercises 1, 2, 3, 7, 8, and 9. While in $v_{CoPy}$, exercises 1, 2, and 3 are the ones with a significant increase in the mean values (*p* < 0.05).

### 3.3. The 95% Prediction Ellipse Area and Statokinesigram

**Figure 12:**
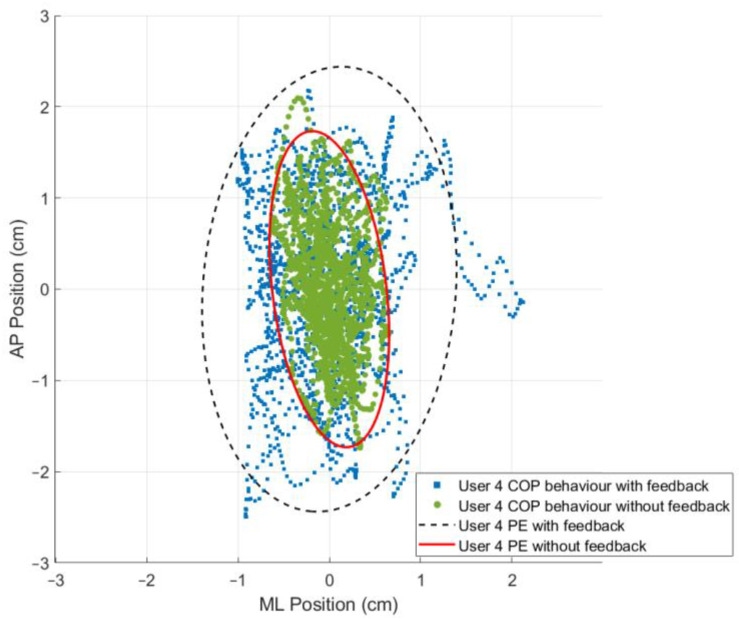
Comparison of the 95% PEAs behaviour with different conditions for exercise 2 ([−5°, +3°] AP; 10°/s).

**Figure 13:**
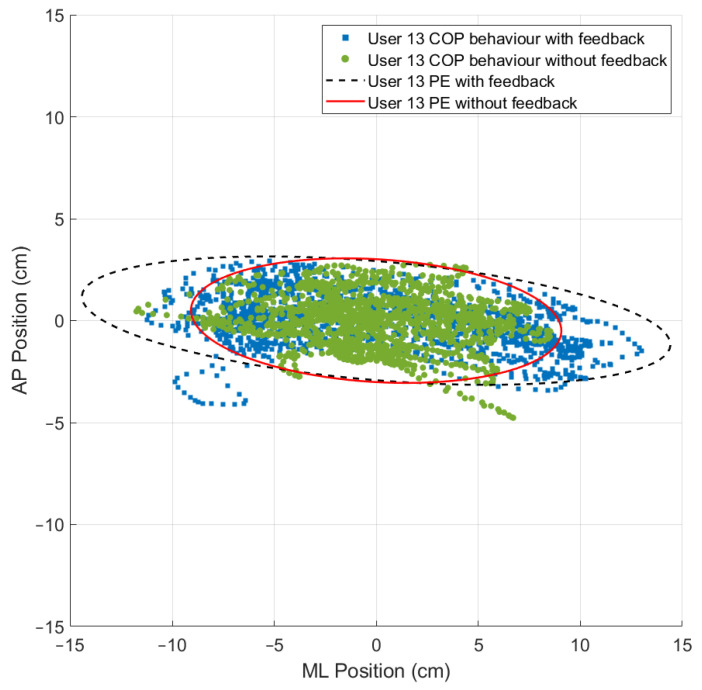
Comparison of the 95% PEAs behaviour with different conditions for exercise 9 ([−4°, +4°] ML; 20°/s).

While using the OREKA platform for assessing human postural control, variations in the 95% PEA provide valuable insights into a user’s balance characteristics. A smaller 95% PEA typically suggests that a user has a more confined movement pattern within the AP and ML directions, indicating better balance stability. Conversely, a larger 95% PEA might imply a more extensive body sway, suggesting that the user may rely on larger compensatory movements to maintain balance (Figure 12 and Figure 13).

**Figure 14:**
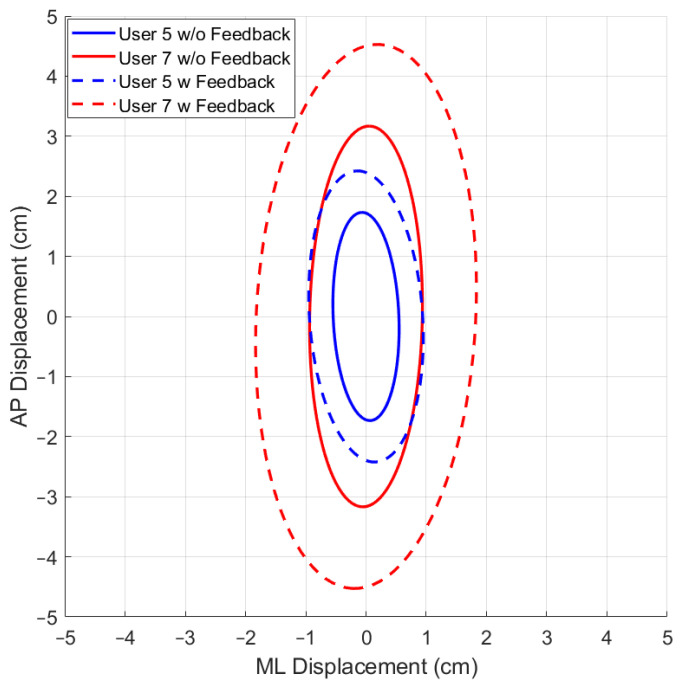
Comparison of 95% PEAs for different users under different conditions for exercise 5 ([−8°, +4°] AP; 10°/s).

**Figure 15:**
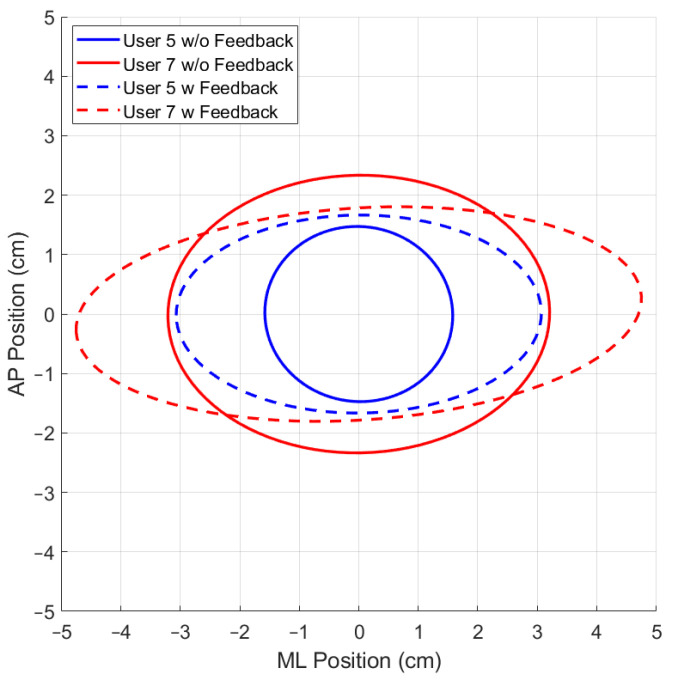
Comparison of 95% PEAs for different users under different conditions for exercise 8 ([−4°, +4°] ML; 10°/s).

By comparing the 95% PEAs of different users, the OREKA platform results help identify individual’s specific postural control strategies, thereby facilitating an approach towards rehabilitation or balance training (Figure 14 and Figure 15).

**Figure 16:**
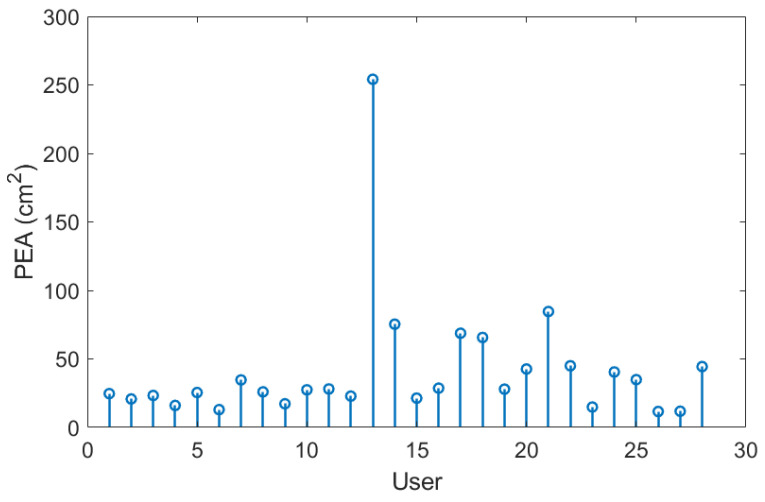
PEAs of the sample for exercise 8 with on-screen feedback ([−4°, +4°] ML; 10°/s).

In addition to the previously mentioned trend, the 95% PEA mean and the standard deviation presented in Table 8 show a significant difference between the areas in the AP and ML rotations. Specifically, the area in the ML rotation tends to be double the magnitude of the AP rotation. This indicates that the CoP signal in the ML direction exhibits more freedom of movement than in the AP direction, further reinforcing the theory that managing balance in the ML direction shows a tendency to be natural to users. Additionally, it is worth highlighting that the large values of the standard deviation in exercises with on-screen feedback further support the use of different postural control strategies by the users when instructed to maintain the markers at the origin. Particularly in exercise 8, where the standard deviation is larger than the mean, the presence of an unusual balance behaviour (outlier) can be identified (Figure 16). It is important to mention that, at this stage of research, we are not discriminating the outliers, but rather determining the balance limits for healthy subjects when using the OREKA platform.

In Table 9, a significant increase in the mean values (*p* < 0.05) is present for exercises 2, 3, 7, and 9.

### 3.4. Poincaré Plot

**Figure 17:**
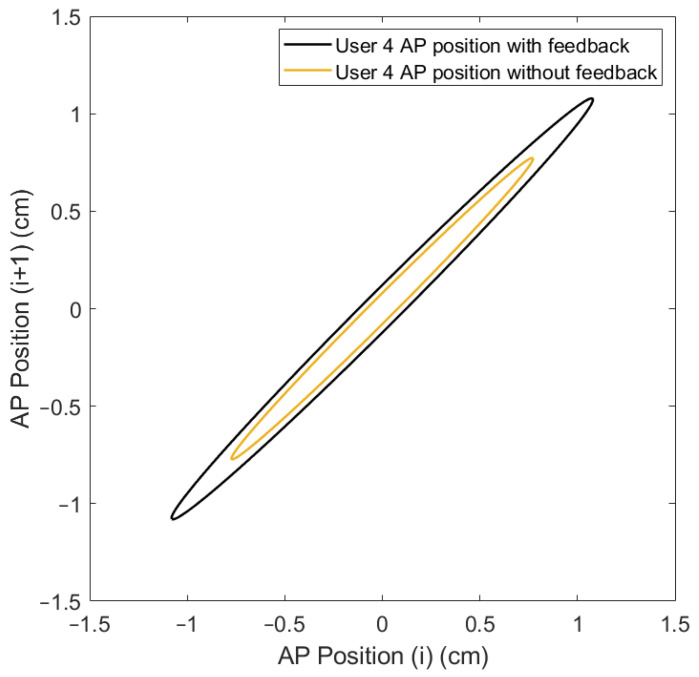
Comparison of Poincaré diagram of AP positions with and without feedback on a screen for user 4.

**Figure 18:**
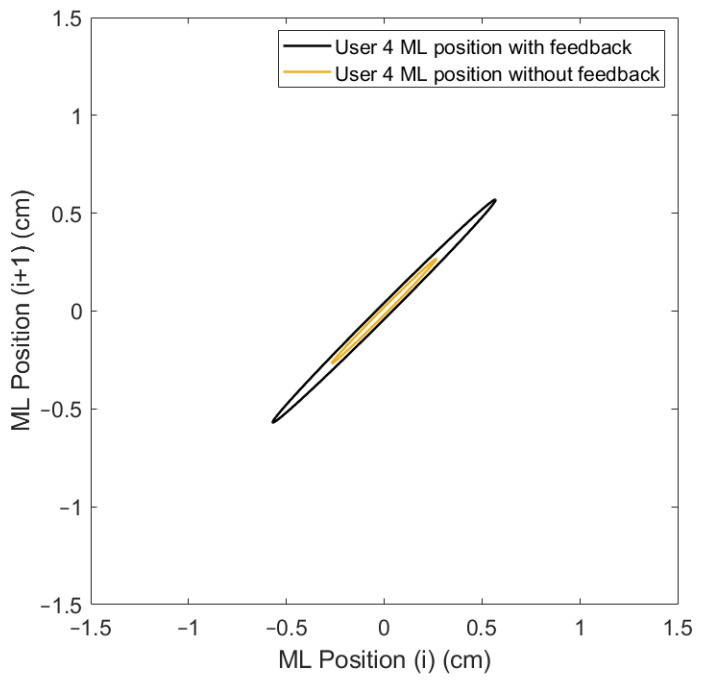
Comparison of Poincaré diagram of ML positions with and without feedback on a screen for user 4.

The variability in the SD1 and SD2 parameters can be visualized by applying the Poincaré diagram representation (Figure 17 and Figure 18) to the ${CoP}_{x}$ and ${CoP}_{y}$ positions presented in the plot of Figure 10. In this work, these parameters were used as reference points to justify the exploration and characterization of the inherent non-linearities in balance systems.

A more compact Poincaré diagram for the test without feedback on a screen compared to the test with feedback on a screen indicates that there is a higher consistency in the CoP signal. This suggests a more regular and predictable postural control strategy in said condition, related to the user’s natural body sway.

**Figure 19:**
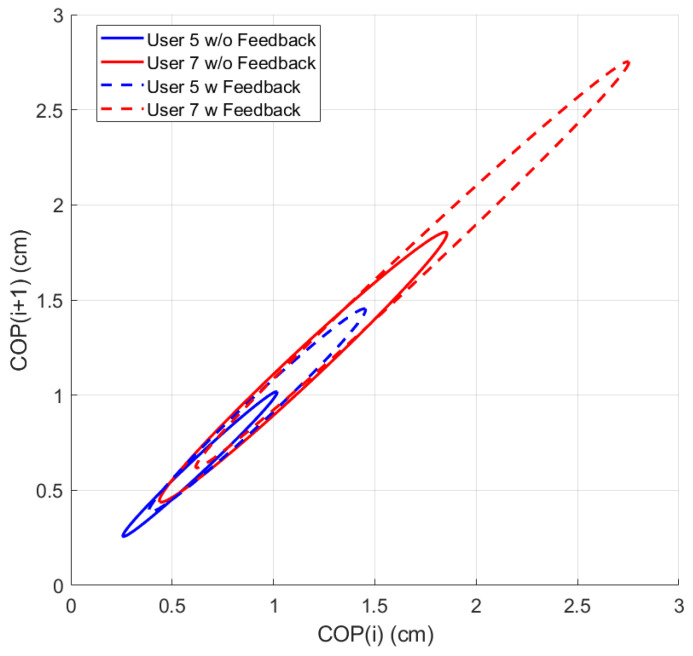
Comparison of Poincaré diagram ellipse behaviour for different users under different conditions for exercise 5 ([−8°, +4°] AP; 10°/s).

**Figure 20:**
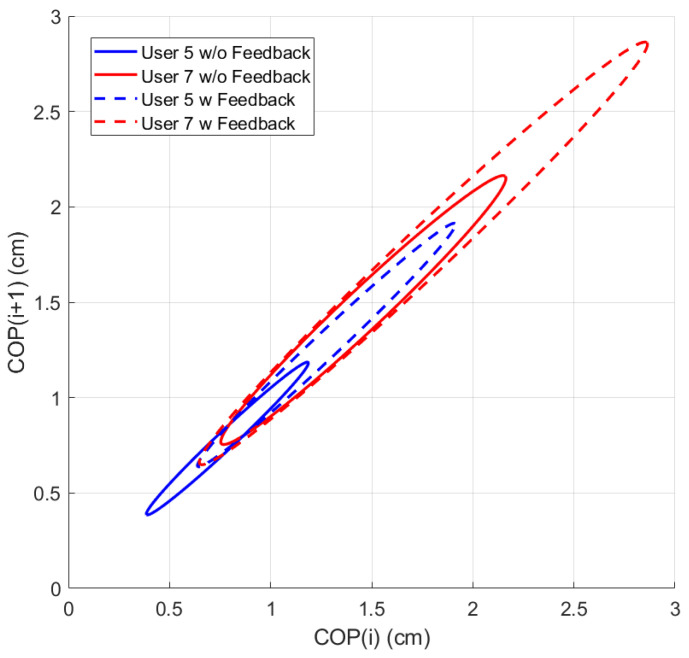
Comparison of Poincaré diagram ellipse behaviour for different users under different conditions for exercise 5 ([−4°, +4°] ML; 10°/s).

The more dispersed Poincaré plot where the condition without feedback on a screen is shown suggests greater variability in the CoP signal. This is representative of users actively controlling their balance, which introduces greater variability in the control strategy as shown in Figure 19 and Figure 20.

In the Poincaré diagram, while the SD1 parameter, as shown in Table 10, exhibits a decrease, it is not significant enough to establish a clear trend. In contrast, a marked decrease in parameter SD2 was noted, particularly when the visual feedback on a screen was removed during the exercises. This reduction indicates a decrease in the long-term variability in the CoP signal, reflecting a more stable and consistent postural control pattern. Interestingly, when analysing the SD2 magnitude in relation to the platform rotation direction, it was found to be higher when the platform was rotated in the ML direction compared to the AP direction. This suggests that a greater dispersion and variability in CoPx body sway is exhibited by the users, indicating a freer movement in the ML direction. These findings further support the theory that the ML direction is perceived as more natural and familiar by users during balance tasks.

In the test conditions with lower amplitude rotations, the increase in velocity coupled with the on-screen visual feedback is significant (*p* < 0.05), as evidenced in the values for SD1 in Table 11 for exercises 1, 2, 3, 7, 8, and 9. Similarly, the values of SD2 highlight a significant increase (*p* < 0.05) for exercises 2, 3, and 9.

## 4. Conclusions

From a general point of view, the data collected from this study provide valuable insights into the influence of feedback on a screen on postural control strategies. When feedback on a screen is removed, a notable reduction in overall positions and velocities is identified, indicating a more adaptive approach to postural control. This response suggests that users rely on natural adjustments to maintain stability during platform rotations. The observed decrease in the prediction ellipse area further supports this notion, indicating a more controlled postural sway without the influence of feedback on a screen.

More specifically, the statistical analysis indicates that tests conducted with low amplitude and high speed, in both ML and AP directions, consistently provide significant outcomes for the selected CoP indices. This suggests that these test conditions are effective in identifying measurable changes in postural control with the conditions of on-screen feedback availability.

Also, it is worth emphasizing that the prototype has enough resolution to find differences not only between tests with and without feedback on a screen, but also between individual participants who, if evaluated by traditional clinical methods, would have obtained the same balance scores. With these results, a more detailed analysis is needed to explore the possibilities in terms of the diagnosis and rehabilitation of people with balance problems.

These findings highlight the importance of considering individual variations when interpreting the adaptive response of postural control. Further research is warranted to explore the specific mechanisms underlying these adaptations and their potential implications for personalized rehabilitation and balance training programs. Understanding how users adapt their postural control strategies without feedback on a screen can provide valuable insights into optimizing interventions for improving postural stability and preventing falls.

## Figures and Tables

**Table 1:** Participant characteristics.

Parameter	Value
Number of participants (*n*)	28
Age (years)	37.5 ± 17.86
Mass (kg)	71.4 ± 12.10
Height (m)	1.72 ± 0.09

**Table 2:** Selected exercises for this study. The first value in the brackets refers to the amplitude of the negative rotations in the anterior (−ψ), and left-side (−ϴ) rotations, respectively. The positive value corresponds to the posterior (ψ) and right-side (ϴ) rotations. The movement always starts from values of ψ=0 and ϴ=0 towards the negative direction.

	$\mathrm{Angular}\;\mathrm{Speed}\;\left(\frac{{}^{\circ}}{s}\right)$		
Amplitude (°)	2	10	20
[−5,+3] AP	E1	E2	E3
[−8,+4] AP	E4	E5	E6
[−4,+4] ML	E7	E8	E9

**Table 3:** CoP indicators.

Indices	Definition	
	ML	AP
Mean distance	$d_{{COP}_{x}}=\frac{1}{N}\sum_{i=1}^{N}\left|{CoP}_{xi}\right|$	$d_{{COP}_{y}}=\frac{1}{N}\sum_{i=1}^{N}\left|{CoP}_{yi}\right|$
Mean velocity	$\frac{1}{N-1}\sum_{i=1}^{N-1}v_{CoPxi}$	$\frac{1}{N-1}\sum_{i=1}^{N-1}v_{CoPyi}$
95% PEA	$PEA=\pi \chi_{0.95,2}^{2}\sqrt{det(S)}$; $S=\left(\begin{array}{cc} s_{x}^{2} & s_{x,y}\\ s_{x,y} & s_{y}^{2} \end{array}\right)$	
Poincaré diagram	$CoP\left(i\right)=\sqrt{{\left(x_{CoPi}\right)}^{2}+{\left(y_{CoPi}\right)}^{2}}$ $CoP\left(i\right)vsCoP\left(i+1\right);$	

**Table 4:** $d_{{CoP}_{x}}$ and $d_{{CoP}_{y}}$ (cm) with (w) and without (w/o) feedback on a screen.

CoP Direction	Feedback	Amplitude	$\mathrm{Angular}\;\mathrm{Speed}\;\left(\frac{{}^{\circ}}{s}\right)$		
			2	10	20
$d_{{CoP}_{x}}$	w	[−5,+3] AP	0.29 ± 0.16	0.40 ± 0.15	0.52 ± 0.17
		[−8,+4] AP	0.31 ± 0.14	0.39 ± 0.17	0.49 ± 0.22
		[−4,+4] ML	1.41 ± 0.81	1.83 ± 1.47	2.20 ± 1.15
	w/o	[−5,+3] AP	0.25 ± 0.12	0.32 ± 0.13	0.42 ± 0.1
		[−8,+4] AP	0.28 ± 0.09	0.35 ± 0.12	0.41 ± 0.15
		[−4,+4] ML	1.36 ± 0.62	1.35 ± 0.83	1.55 ± 0.64
$d_{{CoP}_{y}}$	w	[−5,+3] AP	1.01 ± 0.41	1.07 ± 0.42	1.42 ± 0.59
		[−8,+4] AP	1.30 ± 0.66	1.19 ± 0.52	1.42 ± 0.61
		[−4,+4] ML	0.69 ± 0.19	0.76 ± 0.28	0.79 ± 0.27
	w/o	[−5,+3] AP	0.91 ± 0.55	0.91 ± 0.37	1.13 ± 0.40
		[−8,+4] AP	1.27 ± 0.68	0.96 ± 0.44	1.20 ± 0.47
		[−4,+4] ML	0.55 ± 0.14	0.66 ± 0.17	0.70 ± 0.14

**Table 5:** Percentage of increase and *p*-values for the mean values of $d_{{CoP}_{y}}$ and $d_{{CoP}_{y}}$ (cm) with on-screen feedback with respect to the tests without on-screen feedback.

CoP Direction	Amplitude (°)	$\mathrm{Angular}\;\mathrm{Speed}\;\left(\frac{{}^{\circ}}{s}\right)$					
		2		10		20	
		Increase	*p* Value	Increase	*p* Value	Increase	*p* Value
$d_{{CoP}_{x}}x$	[−5,+3] AP	16.00%	0.3881	25.00%	0.0345	23.81%	0.0186
	[−8,+4] AP	10.71%	0.2830	11.43%	0.2846	19.51%	0.1323
	[−4,+4] ML	3.68%	0.7902	35.56%	0.1447	41.94%	0.0110
$d_{{CoP}_{y}}$	[−5,+3] AP	10.99%	0.4341	17.58%	0.1278	25.66%	0.0301
	[−8,+4] AP	2.36%	0.8365	23.96%	0.0891	18.33%	0.1301
	[−4,+4] ML	25.45%	0.0016	15.00%	0.0884	12.86%	0.1196

**Table 6:** $v_{CoPx}$ and $v_{CoPy}$ (cm/s) with (w) and without (w/o) feedback on a screen.

CoP Direction	Feedback	Amplitude	$\mathrm{Angular}\;\mathrm{Speed}\;\left(\frac{{}^{\circ}}{s}\right)$		
			2	10	20
$v_{{CoP}_{x}}$	w	[−5,+3] AP	0.83 ± 0.34	1.75 ± 0.71	2.76 ± 1.16
		[−8,+4] AP	0.94 ± 0.46	1.65 ± 0.79	2.03 ± 0.86
		[−4,+4] ML	3.63 ± 1.67	8.10 ± 4.54	13.31 ± 6.83
	w/o	[−5,+3] AP	0.70 ± 0.24	1.36 ± 0.52	2.17 ± 0.91
		[−8,+4] AP	0.87 ± 0.43	1.40 ± 0.58	2.01 ± 0.69
		[−4,+4] ML	2.90 ± 0.82	6.03 ± 2.54	9.55 ± 4.15
$v_{{CoP}_{y}}$	w	[−5,+3] AP	2.75 ± 1.22	5.05 ± 1.92	8.81 ± 3.56
		[−8,+4] AP	2.40 ± 0.92	4.59 ± 1.70	7.17 ± 2.74
		[−4,+4] ML	1.32 ± 0.52	2.68 ± 1.57	3.45 ± 2.04
	w/o	[−5,+3] AP	2.07 ± 0.73	4.07 ± 1.50	6.79 ± 2.40
		[−8,+4] AP	2.39 ± 0.80	4.03 ± 1.55	6.10 ± 2.45
		[−4,+4] ML	1.23 ± 0.29	2.16 ± 0.53	2.85 ± 0.88

**Table 7:** Percentage of increase and *p*-values for $v_{CoPx}$ and $v_{CoPy}$ mean values. Velocity (cm/s) with on-screen feedback with respect to the tests without on-screen feedback.

CoP Position	Amplitude (°)	$\mathrm{Angular}\;\mathrm{Speed}\;\left(\frac{{}^{\circ}}{s}\right)$					
		2		10		20	
		Increase	*p* Value	Increase	*p* Value	Increase	*p* Value
$v_{CoPx}$	[−5,+3] AP	18.57%	0.0147	28.68%	0.0234	27.19%	0.0390
	[−8,+4] AP	08.04%	0.5525	17.85%	0.1757	00.99%	0.2065
	[−4,+4] ML	25.17%	0.0417	34.33%	0.0393	39.37%	0.0159
$v_{CoPy}$	[−5,+3] AP	32.85%	0.0041	24.08%	0.0371	29.75%	0.0160
	[−8,+4] AP	00.42%	0.9401	13.89%	0.2105	17.54%	0.1272
	[−4,+4] ML	07.32%	0.4196	24.07%	0.1036	21.05%	0.1596

**Table 8:** The 95% PEAs (cm^2^) with (w) and without (w/o) feedback on a screen.

Feedback	Amplitude	$\mathrm{Angular}\;\mathrm{Speed}\;\left(\frac{{}^{\circ}}{s}\right)$		
		2	10	20
w	[−5,+3] AP	7.69 ± 5.32	13.43 ± 7.22	23.67 ± 14.92
	[−8,+4] AP	11.26 ± 7.60	14.88 ± 10.55	21.75 ± 16.54
	[−4,+4] ML	25.27 ± 12.22	41.13 ± 46.02	48.39 ± 31.10
w/o	[−5,+3] AP	7.30 ± 8.06	9.15 ± 5.85	14.26 ± 7.41
	[−8,+4] AP	9.66 ± 5.61	11.06 ± 7.82	16.06 ± 10.18
	[−4,+4] ML	19.45 ± 7.99	27.2 ± 20.86	33.47 ± 20.33

**Table 9:** Percentage of increase and *p*-values for the mean value of 95% PEAs (cm^2^) with on-screen feedback with respect to the tests without on-screen feedback.

Amplitude (°)	$\mathrm{Angular}\;\mathrm{Speed}\;\left(\frac{{}^{\circ}}{s}\right)$					
	2		10		20	
	Increase	*p* Value	Increase	*p* Value	Increase	*p* Value
[−5,+3] AP	5.09%	0.8311	31.87%	0.0182	39.75%	0.0042
[−8,+4] AP	14.20%	0.3743	25.67%	0.1295	26.13%	0.1275
[−4,+4] ML	23.01%	0.0397	33.87%	0.1504	30.82%	0.0383

**Table 10:** SD1 and SD2 (cm) indices with (w) and without (w/o) feedback on a screen.

PoincaréAxis	Feedback	Amplitude	$\mathrm{Angular}\;\mathrm{Speed}\;\left(\frac{{}^{\circ}}{s}\right)$		
			2	10	20
SD1	w	[−5,+3] AP	0.05 ± 0.02	0.08 ± 0.03	0.13 ± 0.05
		[−8,+4] AP	0.04 ± 0.01	0.08 ± 0.03	0.11 ± 0.04
		[−4,+4] ML	0.06 ± 0.03	0.13 ± 0.09	0.20 ± 0.12
	w/o	[−5,+3] AP	0.03 ± 0.01	0.07 ± 0.02	0.10 ± 0.03
		[−8,+4] AP	0.04 ± 0.01	0.06 ± 0.02	0.10 ± 0.04
		[−4,+4] ML	0.05 ± 0.02	0.09 ± 0.04	0.14 ± 0.07
SD2	w	[−5,+3] AP	0.97 ± 0.35	1.04 ± 0.32	1.27 ± 0.41
		[−8,+4] AP	1.11 ± 0.44	1.20 ± 0.43	1.33 ± 0.50
		[−4,+4] ML	1.28 ± 0.60	1.59 ± 0.79	1.86 ± 0.86
	w/o	[−5,+3] AP	0.81 ± 0.42	0.89 ± 0.31	1.04 ± 0.33
		[−8,+4] AP	0.93 ± 0.37	0.94 ± 0.37	1.13 ± 0.35
		[−4,+4] ML	1.24 ± 0.48	1.24 ± 0.62	1.42 ± 0.55

**Table 11:** Percentage of increase and *p*-values for the mean values of SD1 and SD2 (cm) with on-screen feedback with respect to the tests without on-screen feedback.

Poincare Axis	Amplitude (°)	$\mathrm{Angular}\;\mathrm{Speed}\;\left(\frac{{}^{\circ}}{s}\right)$					
		2		10		20	
		Increase	*p* Value	Increase	*p* Value	Increase	*p* Value
SD1	[−5,+3] AP	66.67%	0.0103	14.29%	0.0210	30.00%	0.0265
	[−8,+4] AP	0.00%	0.8681	33.33%	0.1600	10.00%	0.1601
	[−4,+4] ML	20.00%	0.0369	44.44%	0.0348	42.86%	0.0165
SD2	[−5,+3] AP	19.75%	0.3627	16.85%	0.0270	22.12%	0.0106
	[−8,+4] AP	19.35%	0.3632	27.66%	0.3022	17.70%	0.1199
	[−4,+4] ML	03.23%	0.7538	28.23%	0.1255	30.99%	0.0125

## Data Availability

Data are not available due to privacy restrictions.
